# Scaling Up of Steric Exclusion Membrane Chromatography for Lentiviral Vector Purification

**DOI:** 10.3390/membranes13020149

**Published:** 2023-01-24

**Authors:** Jennifer Julia Labisch, Richard Paul, G. Philip Wiese, Karl Pflanz

**Affiliations:** 1 Lab Essentials Applications Development, Sartorius Stedim Biotech GmbH, August-Spindler-Straße 11, 37079 Göttingen, Germany; 2 Institute of Technical Chemistry, Leibniz University Hannover, Callinstraße 5, 30167 Hannover, Germany; 3 Chemical Process Engineering, Rheinisch-Westfälische Technische Hochschule (RWTH) Aachen University, Forckenbeckstraße 51, 52074 Aachen, Germany

**Keywords:** steric exclusion chromatography, membrane chromatography, scaling up of membrane modules, lentiviral vector purification, polyethylene glycol, depletion potential

## Abstract

Lentiviral vectors (LVs) are widely used in clinical trials of gene and cell therapy. Low LV stability incentivizes constant development and the improvement of gentle process steps. Steric exclusion chromatography (SXC) has gained interest in the field of virus purification but scaling up has not yet been addressed. In this study, the scaling up of lentiviral vector purification by SXC with membrane modules was approached. Visualization of the LVs captured on the membrane during SXC showed predominant usage of the upper membrane layer. Furthermore, testing of different housing geometries showed a strong influence on the uniform usage of the membrane. The main use of the first membrane layer places a completely new requirement on the scaling of the process and the membrane modules. When transferring the SXC process to smaller or larger membrane modules, it became apparent that scaling of the flow rate is a critical factor that must be related to the membrane area of the first layer. Performing SXC at different scales demonstrated that a certain critical minimum surface area-dependent flow rate is necessary to achieve reproducible LV recoveries. With the presented scaling approach, we were able to purify 980 mL LVs with a recovery of 68%.

## 1. Introduction

Lentiviral vectors (LVs) have long been used in the biopharmaceutical industry, primarily in gene-modified cell therapy [1,2]. Stable integration of the LV genome and long-term expression of the transgene have achieved successful therapeutic outcomes for certain diseases, such as acute lymphoblastic leukemia (ALL) [3]. The first pediatric patient with ALL that was treated with LV-based gene-modified cell therapy has now been cancer-free for ten years [4]. In clinical trials, LVs are used to treat a wide range of diseases, including cancers, immune disorders, metabolic disorders, and rare congenital diseases [3,5]. New potential applications for LVs have emerged. Recently, the use of LVs gained importance as a possible vaccination platform that uses integrating as well as non-integrating LVs to target infectious diseases [6,7]. The broad range of diseases that can be treated with LVs and emerging applications will lead to an increased need for efficient LV bioprocessing [8]. Many challenges are faced during LV manufacturing, especially purification (which requires further optimization) [9].

A study on the use of steric exclusion chromatography (SXC) for LV purification was recently published [10]. A variety of viruses have been previously purified by SXC, including baculovirus [11], Orf virus [12,13], AAV [14], and influenza A virus [15]. SXC is a gentle purification method offering high potential for the purification of large, enveloped, fragile viral vectors as it does not require any chemical interaction between the target species and the stationary phase and preserves viral infectivity. The basic principle of SXC is based on depletion interaction [16] and has been discussed in previous publications [10,17,18]. Briefly, the viral vector feed solution is mixed with PEG buffer and loaded onto a hydrophilic stationary phase, e.g., a regenerated cellulose membrane. Upon the addition of PEG, depletion zones around the viral particles and the stationary phase are formed. The resulting depletion interaction results in the association of viral vectors with the stationary phase. The viral particles are eluted with a PEG-free buffer, reserving the association of the viral particles with the stationary phase.

So far, SXC has only been performed at small scales. SXC studies relying on membranes as a stationary phase used stacked membrane layers assembled in their housing (e.g., a stainless-steel holder for multi-use or an overmolded plastic housing for single-use) so that the flow was directed frontally from above, resulting in a dead-end flow [19]. Membrane devices of a diameter between 13 mm and 25 mm with 10 to 20 layers of stacked membranes have been employed in previous publications on SXC [11,12,14,15,20,21,22,23]. However, a deep mechanistic understanding of the requirements of the membrane device is lacking, especially concerning the potential scaling up of SXC. Both the location of viral vector association in the membrane and the effect of different membrane device geometries or sizes on the performance of SXC have yet to be investigated, leaving unanswered questions as to how scaling up could be achieved.

In this study, we show LV location on a stabilized cellulose membrane which served as a stationary phase. Based on these results, we developed a scaling up approach with different device scales and geometries. We reveal the critical aspect of a scaled flow rate, as well as the importance of module design, for successful LV recovery using SXC in a scale-up format.

## 2. Materials and Methods

### 2.1. Lentiviral Vector Production, Harvest, and Clarification

Third-generation lentiviral vectors were produced by transient transfection of suspension HEK293T/17 SF cells (ACS-4500, ATCC) with four plasmids in a UniVessel^®^ 10 L bioreactor operated by a BIOSTAT^®^ B-DCU (Sartorius, Göttingen, Germany). The pH electrode was calibrated, and the vessel was assembled (containing a 2 × 3 blade segment impeller, ring-up sparger) and filled with water equivalent to 30% of its volume. The bioreactor was autoclaved at 121 °C. After autoclaving, the bioreactor was emptied and filled to 80% of the final volume with FreeStyle medium (Thermo Fisher Scientific, Waltham, MA, USA) + 0.0002% Antifoam C (Sigma Aldrich, St. Louis, MO, USA) + 1x insulin-transferrin-selenium (Thermo Fisher Scientific, Waltham, MA, USA). The bioreactor was connected to the BIOSTAT^®^, the DO probe was calibrated, and the pH electrode was re-calibrated. Cultivation setpoints were the following: stirrer speed 202 rpm, 30% DO, 37 °C, pH 7.1. Gassing rates and gassing cascades are given in the supplementary section. The bioreactor was left overnight to adjust pH and pO2. The next day, the bioreactor was inoculated with 9% of the final bioreactor volume to a final viable cell density of 0.3 × 10^6^ cells·mL^−1^. After inoculation, and once daily onwards, the bioreactor was sampled for viable cell density and viability determination with a Cedex HiRes (Roche, Basel, Switzerland) and offline pH measurement. The pH probe was re-calibrated when a difference of >0.1 was detected between the externally and internally measured pH. Three days after inoculation, transfection was performed. Subsequently, 0.5 mg of total plasmid DNA was used per liter of final culture volume in a mass ratio of 5:2.5:1:1 (pALD-Lenti-GFP:pALD-GagPol:pALD-VSV-G:pALD-REV1; Aldevron, Fargo, ND, USA) and was prepared in FreeStyle medium without additives. In a separate flask, 4 mL of PEIpro per mg of total plasmid DNA was diluted in FreeStyle medium (5% of the final bioreactor volume each). The two solutions were mixed and, after incubation for 15 min, added to the bioreactor. The following reagents were added to the bioreactor 18 h after transfection: an anti-clumping agent (1:500 (*v*/*v*)), the enhancer sodium butyrate (final concentration of 10 mM, Sigma Aldrich, St. Louis, MO, USA), and 1 mL of 2% Antifoam C. A nuclease treatment for nucleic acid digestion was performed with 10 U·mL^−1^ DENARASE^®^ (c-Lecta, Leipzig, Germany) and 2 mM MgCl_2_ (final concentrations) directly in the bioreactor for 1 h at 37 °C. After nucleic acid digestion, the cell culture broth (which contained the lentiviral vector) was clarified using Sartoclear Dynamics^®^ Lab V50 (0.45 µm polyethersulfone membrane version) with 5 g/L of diatomaceous earth (Sartorius, Göttingen, Germany). The lentiviral vector was aliquoted and stored at −80 °C.

### 2.2. Steric Exclusion Chromatography

#### 2.2.1. Membrane and Housing

**Figure 1:**
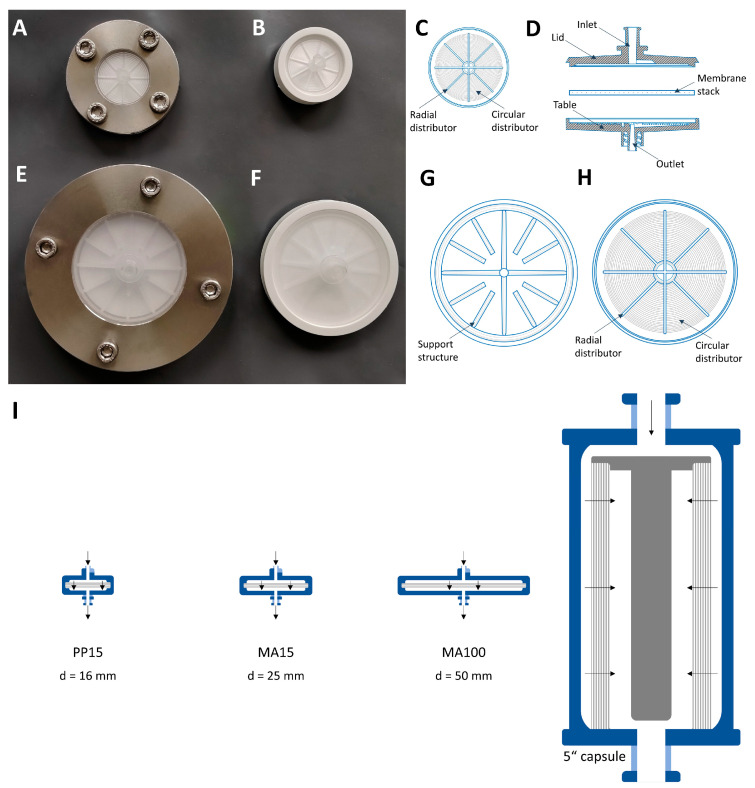
SXC membrane devices. MA15 in a (**A**) stainless-steel holder or (**B**) overmolded. (**C**) The inner structure of the MA15 lid and table with radial and circular distribution channels and (**D**) a cross-sectional view of an SXC membrane device. MA100 in a (**E**) stainless-steel holder or (**F**) overmolded. (**G**) Inner support geometry of the MA100 lid and (**H**) structure of the MA100 table with radial and circular distribution channels. (**I**) Device size comparison of the scale-up approach of SXC using 4 different modules.

An uncharged stabilized cellulose membrane Hydrosart^®^ 10242 (Sartorius, Göttingen, Germany) was used as a stationary phase. For crosslinking, diglycidyl ethers were used as described in detail in [24]. Crosslinking leads to a change in the chemical nature and thus the properties of the membrane, in particular the swelling properties. Pure regenerated cellulose membranes adsorb about 16% of water and thus change its expansion by about 16%. After crosslinking, swelling is reduced by more than half. As a result, the membrane is easier to install and use in the device. Membrane production, characterization, and integrity testing of membrane devices have been previously described by Labisch et al. [10]. The membrane lot used in this study had a thickness of 220 µm per layer and a mean flow pore size of 2.5–3 µm. Stacks of 5 membrane layers were incorporated into the respective polypropylene module housing and either overmolded with an Arburg 221-75-350 injection molding machine or incorporated into a stainless-steel holder so that membranes could be accessed easily during LV visualization experiments (Section 2.3.6). The recommended maximum pressure for axial devices is 0.6 MPa (0.4 MPa for the radial 5′′ device). SXC devices are shown in Figure 1, and specifications are listed in Table 1.

The membrane housing of the MA100 used in this study has a lid and a table with distinct geometries. The lid has a coarse structure with thicker bridges that prevent the membrane from pressing tightly against it which is intended to give the liquid room to spread (Figure 1G). The table has 8 radial distribution channels and 20 circular distribution channels that collect the fluid toward the outlet (Figure 1H). The PP15 and MA15 devices have the same distribution channel geometry in the lid and table (Figure 1C).

#### 2.2.2. Chromatography Setup and Procedure

An ÄKTA™ avant 150 (Cytiva Life Sciences, Uppsala, Sweden) chromatography system with inline UV (280 nm) and conductivity monitoring operated by UNICORN 7.1 software was used to purify the lentiviral vectors via SXC using the PP15, MA15, and MA100 modules. For the large-scale SXC experiments with the 55′′ capsule (Sartorius, Göttingen, Germany) and 4 mm bed height (approx. 18 layers), a multi-use rapid cycling chromatography system (MU RCC, Sartorius, Göttingen, Germany) was used with a PuraLev^®^ i30SU pump (Levitronix, Zürich, Switzerland) installed inline that was operated at 600 rpm, which served as a dynamic mixer for the buffers and feed solution. All chemicals (Tris, hydrochloric acid (HCl), sodium chloride (NaCl), PEG 4000) were purchased from Carl Roth (Karlsruhe, Germany). Buffers were prepared in ultrapure water from the Arium^®^ Pro (Sartorius, Göttingen, Germany). Two buffers were prepared: (1) a 50 mM Tris-HCl buffer with 150 mM NaCl, pH 7.4 (A1), and (2) 25% PEG 4000 in 50 mM Tris-HCl, 150 mM NaCl, pH 7.4 (B1). In the following section, the buffers are referred to as Tris buffer and PEG buffer. The same buffer compositions were used for all experiments based on the buffer optimization experiments previously published [10].

The volumes for equilibration, loading, wash, and elution for all device scales are listed in Table 1. On the day of the experiment, the LV sample was thawed in a water bath at 37 °C until only small ice clumps remained. The sample was then stored at 4 °C until use (30–60 min). The entire LV solution was used on the day of thawing. Different LV batches were used for different experiments; therefore, the respective titer of each LV sample is indicated in the results section. The LV solution was kept on ice during the experiments and the fractions were collected and cooled at 4 °C (automatic fractionation with the ÄKTA avant and manual fractionation with the MU RCC). The membrane device was first equilibrated with the Tris buffer and the PEG buffer, which were mixed inline at a ½ dilution. The PEG buffer with a concentration of 25% (*w*/*v*) PEG 4000 then reached a final PEG concentration of 12.5%. The LV sample (A2) was loaded by being mixed inline with the PEG buffer at a ½ dilution in the downflow direction. The loading volume varied between experiments with the MA100 device and is therefore provided in the results section for each experiment. The membrane was washed with Tris buffer and PEG buffer, which were mixed inline at a ½ dilution. The LVs were eluted with Tris buffer in the upflow direction. Fractions were aliquoted and stored at −80 °C for analysis. The flow rates and SXC membrane device design varied depending on the experiment and are mentioned in the respective results section. To perform SXC experiments with the ÄKTA chromatography system at high flow rates (<10 mL·min^−1^), an open configuration was used for the chromatography system. In this open configuration the fractions were manually collected directly after the installed chromatography device, without running through the whole system to reduce the pressure. A new membrane device was used for each run (single use).

### 2.3. Analytics

#### 2.3.1. Infectious Titer Determination

The infectious LV titer was quantified with the Incucyte^®^ S3 live-cell analysis system (Sartorius, Göttingen, Germany). Adherent HEK293T cells (ACC 635, DSMZ) were infected with serially diluted LV samples, and GFP expression was measured through real-time imaging as described in detail by Labisch et al. [25] with the following modifications: no staining was performed and transgene expression (GFP) was read out 48 h post-infection. Samples were analyzed in duplicate.

#### 2.3.2. Particle Titer Determination

The LV particle titer was quantified with an enzyme-linked immunosorbent assay (ELISA) using the QuickTiter™ Lentivirus titer kit (Cell Biolabs, San Diego, CA, USA) that quantifies the p24 capsid protein. Absorbance was read at 450 nm with a FLUOstar Omega plate reader (BMG Labtech, Ortenberg, Germany). The standard curve obtained was fitted by a second-degree polynomial. The p24 concentrations determined were converted into viral particle titers by assuming that 1.25 × 10^7^ LV particles contain 1 ng of p24 and 1 LV particle contains about 2000 molecules of p24 [26].

#### 2.3.3. Total Protein Quantification

Total protein concentration was determined with the Pierce™ Coomassie Bradford protein assay kit (Thermo Fisher Scientific, Waltham, MA, USA) according to the manufacturer’s instructions. Standards and samples were analyzed in duplicate in transparent 96-well microtiter plates (Greiner Bio-one, Kremsmünster, Austria). Absorbance was read at 595 nm with a microplate reader. The standard curve obtained was fitted by linear regression.

#### 2.3.4. Total dsDNA Quantification

The total dsDNA amount (host cell and plasmid DNA) was quantified with the Quant-iT™ Pico-Green™ dsDNA assay (Thermo Fisher Scientific, Waltham, MA, USA) according to the manufacturer’s instructions. Standards and samples were analyzed in duplicate in black 96-well microplates (Corning, Corning, NY, USA). The samples were excited at 480 nm, and fluorescence emission intensity was measured at 520 nm using a microplate reader. The standard curve obtained was fitted by linear regression.

#### 2.3.5. SDS-PAGE and Silver Staining

Proteins were fractionated by SDS-PAGE in 4–15% Mini-PROTEAN^®^ TGX Stain-Free protein gels (Bio-Rad, Hercules, CA, USA). SDS-PAGE was performed according to the manufacturer’s instructions. Precision Plus protein standard (Bio-Rad) served as a marker. The gel was run at a constant voltage of 300 V for 15–20 min. Protein bands were visualized with a Pierce Silver Stain Kit (Thermo Fisher Scientific, Waltham, MA, USA).

#### 2.3.6. Lentiviral Vector Visualization

Staining was performed to visualize the location of the LVs on the membrane before and after elution. The LV sample was incubated for 1 h at 4 °C with a mouse monoclonal antibody to VSV-G (F-6) labeled with Alexa Fluor^®^ 546 (Santa Cruz Biotechnology, Santa Cruz, CA, USA) in a dilution of 1:2000. Five layers of the Hydrosart^®^ membranes were placed between the table and lid of the chromatography device that was incorporated in the stainless-steel holder (Figure 1A,E). The screws were tightened to 3 Nm. The SXC run was performed as described above and stopped after the wash step before elution. The membranes were separated and visualized with a UVP ChemStudio (Analytik Jena, Jena, Germany) by applying the green light source (550 nm), the ethidium bromide filter, and an exposure time of 60 s. An untreated membrane layer that was not incorporated into the membrane holder device was used as a negative control.

### 2.4. Statistical Analysis

The statistical significance of between-group differences was evaluated using an unpaired Student’s *t*-test (two-tailed) in OriginPro^®^ 2021 (OriginLab, Northampton, MA, USA). Where applicable, experiments were evaluated with MODDE Pro 13 (Sartorius, Göttingen, Germany).

## 3. Results and Discussion

### 3.1. Lentiviral Vector Visualization on a Membrane

To date, it is unclear where the target product (in this case, the LV) is located on the membrane after loading by SXC, and the literature lacks studies of particle localization in the stationary phase during SXC. Visualizing LVs on the stationary phase could contribute to the understanding of the SXC process and process requirements.

**Figure 2:**
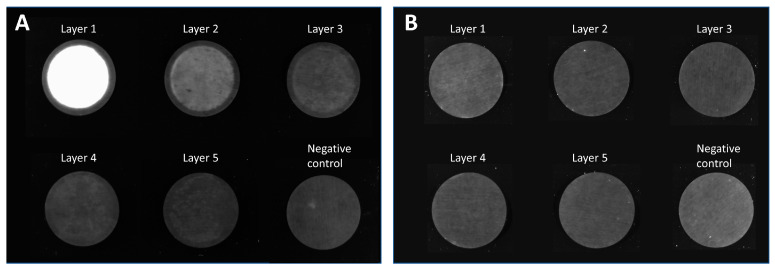
Membrane layers of an MA15 device after loading lentiviral vectors labeled with an anti-VSV-G Alexa Fluor^®^ 546 antibody. Membrane layers were separated after the (**A**) loading and wash step of steric exclusion chromatography or (**B**) after the elution step. A membrane that was not incorporated into the device served as a negative control.

We stained lentiviral vectors with an anti-VSV-G antibody labeled with Alexa Fluor^®^ 546 as described in Section 2.3.6. The labeled LVs were loaded on a membrane that was incorporated into an MA15 housing and placed in a stainless-steel holder (Figure 1A). SXC was performed using a PEG buffer with a final PEG 4000 concentration of 12.5% and a flow rate of 7 mL·min^−1^. A volume of 50 mL was loaded, corresponding to a 25 mL LV solution with 1.5 × 10^11^ VP·mL^−1.^ The SXC runs were stopped either after the loading and wash step (Figure 2A) or after the elution step (Figure 2B) for optical visualization.

Figure 2 shows the visualization of captured viral vectors on the membrane after being loading by SXC. LV particles were mainly present on the first and second layers of the membrane. Some LV particles can be detected on layer three, but no fluorescence was detected on layers four or five. The particles were homogeneously distributed on the membrane layers. Only the clamping edge, which is not in contact with the liquid, was not stained accordingly. These findings indicate that with SXC, we capture very few viral particles in the depth of the unit. Therefore, using 15 layers—as is often described in the literature [11,15]—does not appear to offer any added value compared to the use of 5 layers. Furthermore, column volume, which is specified for other conventional membrane chromatography devices, plays a minor role in the SXC method. Although it is a straightforward approach, visualization of the viral vectors on the membrane indicates that adding more membrane layers (thereby increasing the membrane volume) does not appear to be a valuable scaling method. The surface area of the first layer is a more important feature for SXC. We assume that once the first layer is saturated, the access to further layers is restrained, and a multilayer of LV particles is built that reduces pore size. Thus, a pressure increase is observed during loading, as previously reported [10,22]. After elution, no fluorescence was detected on the membrane, indicating that (almost) all LVs were eluted.

### 3.2. Identifying Critical Process Parameters for the Scaling Up of SXC

**Figure 3:**
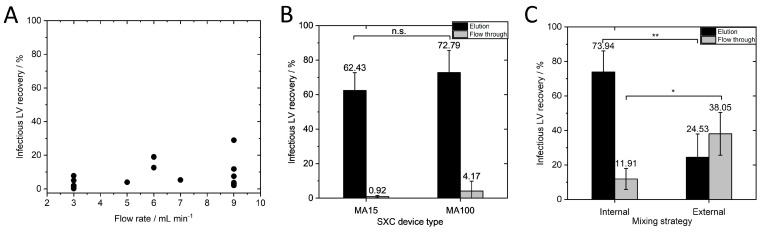
Identification of critical process parameters to achieve a high infectious LV recovery for SXC scale-up. (**A**) Infectious LV recovery for different flow rates between 3 and 9 mL·min^−1^. (**B**) Infectious LV recoveries of MA15 and MA100 with the same surface area-dependent flow rate of 1.43 mL·min^−1^·cm^−2^ (MA15 N = 6, MA100 N = 11). (**C**) Infectious LV recovery for internal and externally mixed LV-PEG-solution, N = 3. Data in B and C represent mean ± standard deviation. *p*-values are indicated as follows: * *p* ≤ 0.05, ** *p* ≤ 0.01, n.s.—not significant.

SXC has so far only been performed at small scales using axial membrane devices with a diameter of up to 25 mm. By increasing the membrane surface area four-fold (MA100 module), we aimed to identify critical process parameters for successful scaling up of the purification of lentiviral vectors via SXC. Previous research with a small-scale MA15 device determined 12.5% PEG 4000 as an ideal buffer for the purification of LVs with SXC [10]. Therefore, this buffer composition was used and not further modified in the following experiments. In the same study using the MA15, an optimal flow rate of 6–7 mL·min^−1^ (tested flow rate range 3–9 mL·min^−1^) was identified, achieving infectious LV recovery above 80%. In a first attempt, we tested flow rates between 3–9 mL·min^−1^ using the MA100 device and an LV batch with a titer of 1.25 × 10^7^ TU·mL^−1^. Figure 3A shows that lower than expected infectious LV recoveries were observed. We concluded that the optimal flow rate for the MA100 device is not within this range.

For membrane chromatography, the flow rate is typically given in membrane volumes per minute. As discussed in Section 3.1, scaling up by only increasing the membrane volume but not the surface area of the first membrane does not seem to be useful with respect to the surface-oriented capture of the vector particles. Thus, specification of the flow rate per membrane surface area (of one layer) would be more reasonable than flow rate per membrane volume. For this reason, we did not scale flow rate with membrane volume as a first attempt. It was shown in a previous study that scaling the flow rate according to membrane volume is not necessary for axial devices with the same diameter [10]. In the aforementioned study, MA15 devices with 5 and 10 membrane layers achieved LV recoveries that were not significantly different when applying the same flow rate of 7 mL·min^−1^, which is the same surface area-dependent flow rate of 1.43 mL·min^−1^·cm^−2^. It should be noted that scaling with the surface-area dependent flow rate was still unknown and not discussed in the previous study, as no different device sizes were tested. This parameter is investigated in our study for the first time. However, 7 mL·min^−1^ was half the flow rate in membrane volumes per minute for the 10-layer unit compared to the 5-layer unit (6.2 MV·min^−1^ for the 10-layer MA15 and 12.4 MV·min^−1^ for the 5-layer MA15). If flow rate had to be scaled with membrane volume, this would have been noticed in the experiment, and the non-significant differences indicated that this was not necessary. For this reason, the flow rates for the MA100 device were not adjusted according to membrane volume, though the same volumetric flow rates were used since an adjustment based on the membrane area of the first layer was only considered in the next step. Given the dynamic depletion flocculation process of SXC, we hypothesized that the flow rate is dependent on the surface area of one membrane layer. The previously determined optimal flow rate of 7 mL·min^−1^ for the MA15 device corresponds to a surface area-dependent flow rate of 1.43 mL·min^−1^·cm^−2^. We aimed to apply the same surface area-dependent flow rate for the MA100 device. As the membrane surface area of one layer is four times larger, a flow rate of 1.43 mL·min^−1^·cm^−2^ for the MA100 device corresponds to 28 mL·min^−1^.

However, the pressure limit was reached when applying 28 mL·min^−1^ with the viscous PEG buffer. The UV cell and fractionator of the chromatography system contribute to the pressure. To circumvent this technical limitation, we opened the chromatography system after the column position and fractionated manually. This adjustment allowed us to apply a flow rate of 1.43 mL·min^−1^·cm^−2^ (28 mL·min^−1^) for the MA100 device. We performed SXC runs with an LV batch with a titer of 1.73 × 10^7^ TU·mL^−1^ at 1.43 mL·min^−1^·cm^−2^ with the MA15 and MA100 device and detected no significant differences in infectious titer (Figure 3B). The MA100 yielded an infectious LV recovery of 72.79 ± 12.92%. These results confirm our hypothesis that the flow rate must be scaled to the surface area of one membrane layer. The flow velocity through the stationary phase seems to be a decisive factor in purification success. When the same flow rate in mL·min^−1^ is applied to the MA100 device, the same feed is distributed to a larger surface area and, thus, to a higher number of pores compared to the MA15 device. Since the average pore diameter remains unchanged, the flow velocity inside the pores decreases and falls below the optimal flow velocity inside the stationary phase to achieve efficient LV capture during loading and release during elution. Another possible explanation, recently discussed in [13], is that a limited spontaneous encounter for the LV and the stationary phase could lead to a less efficient depletion interaction. In our case, a certain flow rate through the membrane pores might be necessary to increase the probability of an encounter between the LVs and the stationary phase.

Internal and external mixing of the LV solution with PEG buffer was performed for the MA100 SXC runs as was previously performed for the MA15 runs [10]. Briefly, LV solution (titer of 1.64 × 10^7^ TU·mL^−1^) was mixed with PEG buffer in a flask with a magnetic stirrer. After 1 h of incubation at 4 °C, the sample was loaded onto the membrane (external mixing) or was loaded via pump A and pump B of the chromatography system and mixed in the dynamic mixer shortly before reaching the membrane device. Infectious LV recovery was significantly higher (*p* ≤ 0.01) when internal mixing was performed (Figure 3C) than when external mixing was performed (73.94 ± 12.13% and 24.53 ± 13.43%, respectively). These findings support the results of Labisch et al. and Eilts et al., in which the same effect was observed for other module sizes [10,13]. Moreover, a significantly higher amount of LVs (38.05 ± 12.37%) was lost in the flow through (*p* ≤ 0.05) when LVs were loaded after external mixing. The external mixing of PEG buffer with the LV solution and incubation could have led to LV aggregation as depletion interaction can occur between LVs and the stationary phase during the SXC loading step, as well as between the viral particles themselves [27]. Forming aggregates, the system’s free energy is already reduced, leading to a less effective depletion interaction between the LV and the membrane and loss in the flow through. These observations underline the highly dynamic nature of this chromatography method, as already observed by the importance of flow rate.

**Figure 4:**
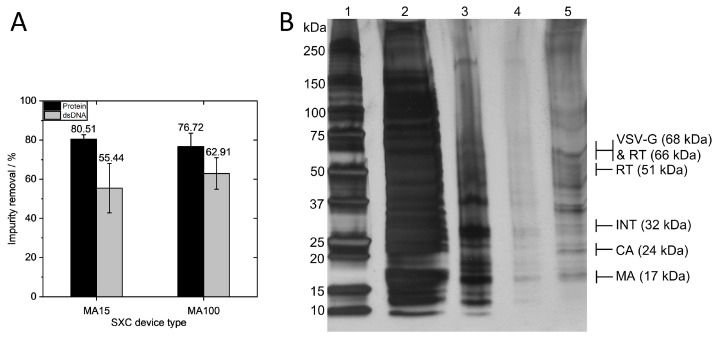
Impurity removal via steric exclusion chromatography. (**A**) Total protein and dsDNA removal using the MA15 and MA100 devices with the same surface area-dependent flow rate of 1.43 mL·min^−1^·cm^−2^ (MA15 N = 6, MA100 N = 11). (**B**) SDS-PAGE gel with silver staining of SXC fractions: 1—marker, 2—loading material, 3—flow through, 4—wash, 5—elution. Protein bands refer to VSV-G envelope protein, reverse transcriptase (RT) subunit p51 and p66, integrase (INT), capsid (CA), and matrix (MA). Data in A represent mean ± standard deviation.

Total protein and dsDNA removal using the MA15 and MA100 devices at the same surface area-dependent flow rate of 1.43 mL·min^−1^·cm^−2^ was next investigated (Figure 4). The total dsDNA and protein concentrations of the loading material and elution fractions are listed in Table 2.

Overall, high removal of proteins was observed, with 80.51 ± 2.22% (0.7 log removal) for the MA15 device and 76.72 ± 6.81% (0.64 log removal) for the MA100 device. A silver-stained SDS-PAGE gel confirmed the measurement, showing a high amount of protein contaminants in the loading material and the removal of the majority of the protein impurities in the flow through (Figure 4B). The elution fraction shows protein bands for the structural proteins of the lentiviral vector and little contaminating protein. Total dsDNA removal was 55.44 ± 12.58% (0.35 log removal) for the MA15 device and 62.91 ± 8.06% (0.43 log removal) for the MA100 device. These results demonstrate that comparable impurity removals are obtained for both device types. The effective removal of impurities derives from the pronounced size differences between the LV and the contaminating proteins and DNA, as discussed extensively elsewhere [10].

**Figure 5:**
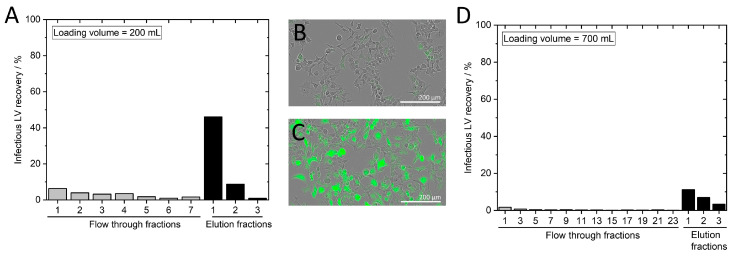
Identification of loading capacity. Infectious LV recovery for loadings of (**A**) 200 mL and (**D**) 700 mL. Phase contrast image merged with a green fluorescence channel image of HEK293T cells after incubation with a (**B**) flow through fraction or (**C**) elution fraction.

Next, we tested different loading volumes ranging from 100 to 700 mL (corresponding to 50 to 350 mL LV solutions) on the MA100 device. The LV batch had a titer of 1.35 × 10^7^ TU·mL^−1^ and 1.14 × 10^10^ VP·mL^−1^. Previous SXC experiments with the MA100 were performed by loading 200 mL. Flow through and elution fractions of all runs were analyzed. No increase in the amount of LVs in the flow through was observed as the loading volume increased, which is exemplarily shown in Figure 5A,D.

These findings are supported by captured images of HEK293T cells expressing no GFP after transduction with the flow through fractions (Figure 5B). In contrast, HEK293T cells transduced with LVs from the elution fractions showed GFP expression (Figure 5C). When a high LV amount was loaded (Figure 5D), the elution of the captured LVs was hardly possible, resulting in a low recovery in the elution fraction. The highest LV recovery was achieved by loading around 200 mL (Figure 5A). Therefore, we define a loading capacity of 1.35 × 10^9^ TU and 1.14 × 10^12^ VP. In contrast to conventional chromatography methods, SXC does not rely on a stationary phase having functional groups, and thus limited binding sites, which typically results in a breakthrough that is observed once all binding sites are occupied. During our SXC runs, no LV breakthrough was observed. Thus, membrane capacity for SXC cannot be defined at 10% LV breakthrough; instead, different loading volumes and the success of LV elution are analyzed to determine the loading capacity at which the LV recovery in the elution is satisfactory. In the previous experiments (Figure 3B), 4.10 × 10^8^ TU and 1.60 × 10^9^ TU were loaded onto the MA100 and MA15 device, respectively, showing that approximately four times as many LVs could be loaded onto the MA100 device compared to the MA15 device. The loaded amount of LVs was lower than in the previous study, in which CAR-T-based LVs were used with a higher LV titer in the loading material [10]. These differences in the upstream material are likely the reason for the different outcomes, and it might be necessary to determine the ideal loading volume for each target product separately. Another reason could be the uneven LV distribution on the membrane with the MA100 standard housing discussed in Section 3.3, which might lead to the inefficient elution of overloaded membrane areas.

To further analyze the presented approach of scaling the flow rate according to the membrane surface area of the first layer, we performed scale-down experiments with an axial PP15 device for three different flow rates (N = 3 each) and a scale-up experiment with a radial 5′′ device for two different flow rates (N = 1 each). Additionally, further runs at different flow rates with the MA15 and MA100 modules were performed (N = 3) to complement the data.

According to the literature, this is the first study using a membrane capsule for SXC and includes the largest membrane module that has been used for this method to date, with a loaded LV volume of 0.98 L. Pressure limitation was often discussed as a potential hurdle for SXC scale-up. As previously reported, the viscous buffers result in higher pressure compared to conventional chromatography methods such as anion exchange chromatography, and pressure increases during loading have often been reported [10,22,23]. We observed a pressure increase during the two scale-up runs with the 5′′ capsules of 0.4 to 0.8 bar (run 1) and 0.5 to 0.7 bar (run 2). As the pressure limit of the device is 4 bar, pressure was not a limiting factor during the scale-up runs under the tested conditions.

**Figure 6:**
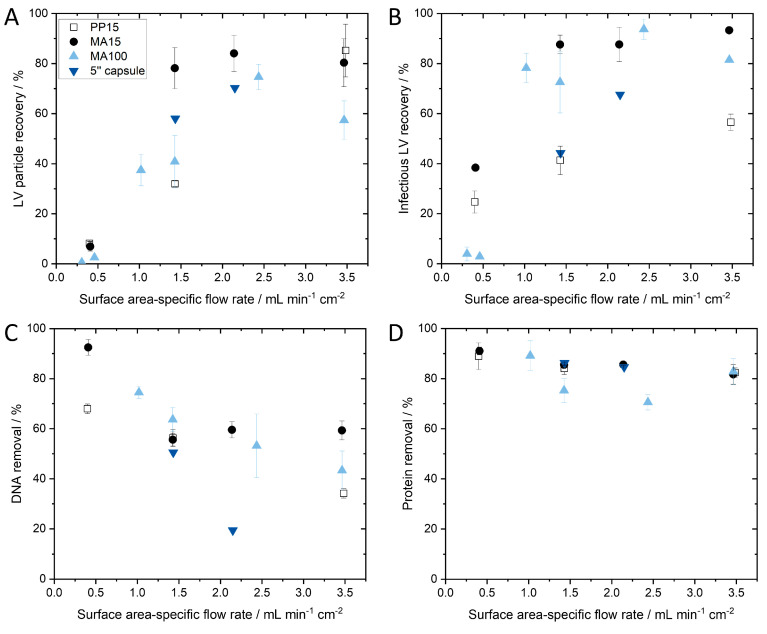
(**A**) Particle and (**B**) infectious LV recovery and (**C**) dsDNA and (**D**) protein removal in SXC experiments plotted against different surface area-specific flow rates for different module sizes. Replicates for each device and flow rate are as follows: PP15, N = 3 for all flow rates; MA15, N = 3 for all flow rates; MA100, for 1.43 mL·min^−1^·cm^−2^ N = 11, for all other flow rates N = 3; 5′′ capsule, N = 1.

The infectious and particle recoveries and impurity removals for the four different device scales are shown in Figure 6 and are plotted against different surface area-specific flow rates.

Plotting the infectious and particle titer recoveries of the tested device scales against different surface-area specific flow rates shows that if the flow rate falls under a critical minimum flow rate, then LV recovery decreases significantly (Figure 6A,B). It appears that LV recovery asymptotically approaches a maximum. A decrease in LV recovery at flow rates above 3.5 mL·min^−1^·cm^−2^ is possible; however, there is a technically feasible limit due to the maximum flow rate of the system and the maximum pressure of the module. Further investigation is necessary to confirm this observation, but it is clear that a surface area-dependent flow rate that is too low significantly reduces LV recovery. In general, a surface area-dependent flow rate of approximately 1.4 mL·min^−1^·cm^−2^ or higher is necessary for successful scaling up of SXC. The reason why a critical minimum flow rate is necessary can be explained when considering the capture mechanism on the membrane. An association (capture) of the LVs on the membrane takes place when the depletion zones of the LVs and the membrane overlap. This occurs through random encounters between the LV and the membrane while passing through the membrane. When the flow rate is increased, the turbulence within the membrane increases as well, which in turn is expected to increase the likelihood of LVs encountering other LVs or the membrane for depletion interaction. We expect that the effect approaches a maximum probability of encounters that can be observed in an asymptotical trend.

When scaling the flow rate and loaded LV volume according to the membrane area of the chromatography module, the processing time for a complete chromatography run remains constant; thus, SXC runs with an MA15 or a 5′′ capsule both take approximately 20 min at 1.43 mL·min^−1^·cm^−2^. This short processing time is especially beneficial for fragile enveloped viruses and viral vectors and enables a fast and efficient DSP process. With the scaling approach of using a minimum surface-area dependent flow rate, we were able to achieve reproducible SXC LV recovery at four different module sizes. The highest LV volume purified by SXC was 980 mL, with a recovery of 68% representing an overall scaling factor of 98 compared to the smallest device (PP15) (Table 1). dsDNA removal shows a decreasing trend with increasing surface area-specific flow rate (Figure 6C). To achieve both high LV recovery and dsDNA removal, a surface area-specific flow rate between 1.4 and 2.5 mL·min^−1^·cm^−2^ is preferred, which subsequently achieves approximately 51% dsDNA removal. Protein removal was unaffected by flow rate and was consistent for the different module sizes, with a protein removal of about 84% (Figure 6D). Good overall impurity removals were achieved and a subsequent ultrafiltration and diafiltration step will likely follow the DSP process to remove residual PEG and further increase the purity of the product.

### 3.3. Influence of the Design of the Membrane Housing on SXC Performance

After identifying critical process parameters for the scaling up of SXC, we investigated the impact of the design of the membrane housing of the MA100 module on LV capture in the membrane and SXC performance.

The membrane chromatography devices used in this study are operated by an axial flow from above through a membrane stack and have a low bed height (height of superimposed membrane layers). Besides a lower bed height, the incident flow area is larger than the resin columns. A uniform flow distribution over the entire membrane area is required to avoid channeling and to enable the whole membrane area to be used efficiently. Even flow distribution is achieved by a distributor structure inside the lid, which spreads the fluid over the membrane, and a collector structure inside the table, which collects the fluid. As housing geometry significantly influences fluid transport through the membrane, housing design should play a major role during chromatography process development [28,29].

Axial devices are limited by their central inlet, resulting in a velocity profile; however, they still have the advantage of simple production and are therefore preferred at small scales.

**Figure 7:**
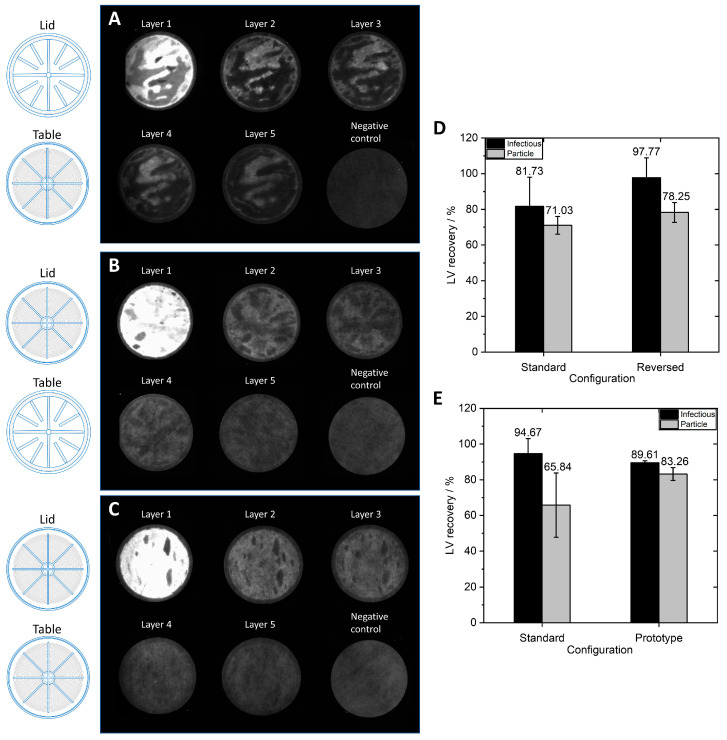
Influence of different MA100 configurations on the location of labeled LVs (anti-VSV-G Alexa Fluor^®^ 546 antibody) on the membrane after loading and on the recovery of infectious and particle LVs. Membrane layers were separated after the loading and washing steps of steric exclusion chromatography. The lid and table configurations of the housing are indicated on the left and include a (**A**) standard configuration, (**B**) reversed configuration, and (**C**) prototype configuration. (**D**,**E**) Infectious and particle LV recovery for the three configurations of the MA100 housing operated at 1.43 mL·min^−1^·cm^−2^ (N = 3 each). Data represent mean ± standard deviation.

Lentiviral vector visualization with the MA100 housing was carried out to assess LV distribution on the membrane before and after elution. SXC was performed at a flow rate of 1.43 mL·min^−1^·cm^−2^. A volume of 160 mL was loaded, which corresponds to a volume of 80 mL of LV solution with a particle titer of 7.27 × 10^9^ VP·mL^−1^ and an infectious titer of 2.71 × 10^7^ TU·mL^−1^. For the first SXC run with labeled LVs, a standard housing configuration was used with the lid and table having distinct structures (Figure 1G,H). Figure 7A displays an uneven distribution on the membrane layers with a consistent appearance throughout all membrane layers. As previously observed with the MA15 device (Figure 2), the LVs are mainly located on the upper layers, although some LVs also reach the bottom membrane layers. The fluid does not seem to have been distributed evenly over the membrane. This uneven fluid distribution has favored membrane channeling and an imbalanced utilization of the membrane layer, thus leading to the overloading of some areas. These overloaded areas can potentially lead to the poorer detachment of particles, causing them to remain aggregated on the membrane.

For the second SXC run, the device’s configuration was reversed; the table (Figure 1H) was used as a lid and vice versa. Therefore, the incoming fluid was distributed by the radial and circular distribution channels. Figure 7B shows that the LVs are more evenly distributed on the membrane layers. LV presence on the first membrane layer is visible, comparable to the findings when using the MA15 module (Figure 2). The changed lid and table configuration in this run highly improved fluid distribution over the membrane. These findings demonstrate that a lid with radial and circular distribution channels is better suited than a coarse structure with thick bridges when seeking to spread the fluid over the membrane (Figure 1G). The dark spots within the bright areas indicate the presence of air bubbles that did not allow the fluid to access the membrane in this area. Air bubbles prevent the utilization of the surface area they occupy, reducing the recovery of target species. A higher pressure drop across the membrane could eliminate air bubbles; alternatively, an optimized module design might be necessary.

A prototype was constructed with radial and circular distribution channels in the lid and the table. This housing configuration also resulted in evenly distributed LVs on the membrane (Figure 7C). Some air bubbles were present in the device (dark spots). Comparing Figure 7B,C, the LVs appear to be better distributed with the prototype housing. A possible reason is that the fluid is not only evenly distributed on top of the membrane stack but is also collected from the membrane more efficiently and directed to the outlet of the table with the distribution channel design. This LV visualization experiment shows that the membrane module’s design is crucial to achieving evenly distributed fluid on the membrane so that the whole membrane area can be utilized.

The three device configurations explained above were used to purify LVs via SXC. For this experiment, the membranes, lid, and table of the MA100 module were incorporated into a stainless-steel holder (Figure 1E). Thus, comparability to the overmolded MA100 devices used for previous experiments is limited. The LV recoveries for different MA100 housing configurations are shown in Figure 7D,E. The standard configuration was used for all previous experiments and served as a comparison for the reversed and prototype configurations. The virus solution purified with the standard and reversed configuration (Figure 7D) had a total particle titer of 1.02 × 10^10^ VP·mL^−1^ and an infectious titer of 2.39 × 10^7^ TU·mL^−1^. The virus solution purified with the prototype device and the standard configuration (Figure 7E) had a total particle titer of 4.32 × 10^9^ VP·mL^−1^ with a concentration of 3.03 × 10^7^ TU·mL^−1^.

The reversed configuration generated higher infectious LV recovery and total LV particle recovery compared to the standard configuration, although differences were not significant. The LV recoveries of the prototype configuration were also not significantly different from the standard configuration, though standard deviations were lower with the prototype configuration. These findings indicate that utilizing a distribution structure on the inlet and outlet side allows for generally more stable reproduction of LV recoveries. The prototype and reversed configuration reduce the dead volume on the inlet side, which decreases back-mixing effects and promotes a narrower residence time distribution [28]. Concerning the high LV recoveries, the uniform LV distribution on the membrane, and the lowest dead volume, the prototype device with a flow distributor plate in the lid and table is the favored configuration for an axial MA100 device. Further device optimizations are necessary to avoid the entrapment of air bubbles in the module. Moreover, other module geometries, such as the capsule format with a radial flow, showed promising results in our study and have the advantage of a homogenous flow distribution that has been previously discussed in several studies [28,30,31] and that can be easily scaled [32].

## 4. Conclusions

Steric exclusion chromatography has been demonstrated to have potential as a gentle purification method for large enveloped viral vectors. However, scaling up has not yet been investigated, raising the question of how to approach this challenge. Visualization of the LVs on the membrane showed that SXC is a surface-oriented process, meaning that LVs are mainly captured on the upper membrane layer. We demonstrated that flow rate must be scaled with the membrane area of the first layer. Scale-down and scale-up experiments demonstrate that a certain critical minimum surface area-dependent flow rate is necessary to achieve reproducible LV recoveries with the four different device scales tested. These devices had an overall scaling factor of 98. For the largest scale runs, a radial device geometry was successfully used to purify 980 mL of LVs, and further scaling up could be realized by using larger capsules or cassette modules. Investigating various loading volumes showed no LV breakthrough with increasing volume. However, the elution of LVs from overloaded membrane areas was hardly possible, indicating an optimal amount of LVs to be loaded. Altering the design of the MA100 module housing improved flow distribution and led to a uniform distribution of LVs on the membrane. The use of improved housing prototypes could offer the possibility of loading more LVs, as overloading of membrane areas is more likely to be avoided. Overall, we have demonstrated the scalability of SXC using membrane modules, providing a basis for potential future industrial applications of the method.

## Figures and Tables

**Table 1:** Specifications for SXC chromatography with different device scales.

Specification	PP15	MA15	MA100	55′′ Capsule
Equilibration volume	8 mL	20 mL	80 mL	2 L
Loading volume	20 mL	40 mL	160 mL or higher	1.96 L
Loaded LV volume	10 mL	25 mL	80 mL or higher	0.98 L
Wash volume	8 mL	15 mL	60 mL	2 L
Elution volume	10 mL	20 mL	80 mL or higher	0.98 L
Device geometry	axial	axial	axial	radial
Diameter of axial devices	16 mm	25 mm	50 mm	-
Surface area per layer	2.01 cm^2^	4.91 cm^2^	19.63 cm^2^	192 cm^2^
Membrane volume	0.22 cm^3^	0.565 cm^3^	2.257 cm^3^	75 cm^3^
Scaling factor	1	2.5	10	98

**Table 2:** Total protein and dsDNA concentration in loading material and elution fractions for SXC chromatography with the MA15 or MA100 device.

Device	Protein in Loading Material/µg·mL^−1^	Protein in Elution Fraction/µg·mL^−1^	dsDNA in Loading Material/ng·mL^−1^	dsDNA in Elution Fraction/ng·mL^−1^
MA15	268.45 ± 10.06	52.26 ± 5.65	283.49 ± 87.07	55.44 ± 12.58
MA100	253.38 ± 39.95	57.38 ± 15.15	356.35 ± 81.43	62.91 ± 8.06

## Data Availability

Not applicable.
